# How does a lifetime of painful experiences influence sensations and beliefs about pain in adults with severe haemophilia? A qualitative study

**DOI:** 10.1080/09638288.2021.2018053

**Published:** 2021-12-24

**Authors:** P. McLaughlin, M. Hurley, P. Chowdary, D. Stephensen, K. Khair

**Affiliations:** a Faculty of Health, Social Care and Education, St George’s University of London and Kingston University, London, UK; b Katharine Dormandy Haemophilia and Thrombosis Centre, Royal Free Hospital, London, UK; c East Kent Hospitals University NHS Foundation Trust, Canterbury, UK; d Centre for Outcomes and Experience Research in Child Health, Illness and Disability (ORCHID) Research Unit, Great Ormond Street Hospital for Children NHS Trust, London, UK; e Haemnet, London, UK

**Keywords:** Haemophilia, pain, qualitative research, experiences and beliefs, identity, reflexive thematic analysis

## Abstract

**Purpose:**

To explore the life experiences of pain in people with severe haemophilia and understand how such experiences influence beliefs and sensation of pain in adulthood.

**Methods:**

A qualitative inquiry approach using focus groups and semi-structured individual interviews was used. Participants included people with severe haemophilia living with chronic pain. Data were analysed using reflexive thematic analysis.

**Results:**

Fourteen men with a median age of 47 (range 23–73) agreed to take part. Eleven participated in two focus groups and three were interviewed over telephone. Two themes were conceptualised from the data: (i) haemophilia and pain – an evolving life biography (the personal narrative, historical, social, and medical context, continuous adaptation of activity choices, surveillance of pain and its meaning); (ii) “*My normal isn’t normal*” – identity and self-agency (pain as a feature of life and identify with severe haemophilia, loss of enjoyable activities balanced against staying active, barriers to participation).

**Conclusions:**

Pain is a constantly evolving, lifetime feature for many adults with haemophilia and it is viewed as part of their identity with their condition. Healthcare professionals working in haemophilia should try to better understand the influence of an individuals lived experience with their haemophilia on beliefs and behaviours of pain.

**Implications for rehabilitation**
- Severe haemophilia is a rare bleeding disorder that results in musculoskeletal joint disease.
- Adults with severe haemophilia have experienced multiple episodes of bleeding related musculoskeletal pain since childhood.
- Pain beliefs and behaviours in adulthood appear to be influenced by a lifetime of painful experiences associated with haemophilia.
- In order to better support people with haemophilia and chronic pain, healthcare professionals in haemophilia need to better understand how an individuals lived experience of pain helps inform their beliefs about it.

## Introduction

Haemophilia A and B are rare X-linked congenital bleeding disorders, where normal circulating levels of clotting proteins (factors VIII and IX) that help maintain adequate haemostasis are significantly reduced or absent [1]. The World Federation of Hemophilia estimates a prevalence of 17.1/100 000 for haemophilia A and 3.8/100 000 for haemophilia B. Globally, it is expected that approximately 794 000 people have haemophilia, with 34% being severe (that is a factor VIII or IX level less than 1% of normal) [2]. In the UK, there are 8248 people registered with haemophilia, of which 2145 are severe [3].

Recurrent articular bleeding is a hallmark of haemophilia and is one of the most disabling features of the condition, with bleeding occurring early in the life of those affected, and continuing throughout the lifetime if the condition if left untreated [4]. Management of severe haemophilia involves regular intravenous replacement of the missing clotting factor or other more novel non-factor therapy to prevent spontaneous bleeding [5]. Haemophilic arthropathy is the consequence of repeated joint bleeding and is characterised by chronic synovitis and cartilage destruction, epiphyseal enlargement and bony deformity [6]. Many PWH over the age of 65 had no access to regular treatment until they were in adulthood, with those currently aged in their 40s having no access to effective treatment for the majority of their childhood. Consequently, many PWH have chronically painful, multi-joint haemophilic arthritis [7].

Pain is a cardinal sign of acute musculoskeletal bleeding episodes [8], whereas chronic pain associated with HA is now considered a significant clinical comorbidity. Pain is highly prevalent in PWH with data reporting acute pain being experienced by 20–68% [9,10] whilst chronic pain is present in between 30 and 71% of adults [10,11]. Whilst the basic science investigating pain mechanisms in PWH is lacking, it is generally accepted that both acute and chronic nociceptive input plays a large part in the pain experience in this population [12], with more recent data suggesting the possibility of neuropathic mechanisms in a smaller subset [13,14]. Intra-articular iron deposition following bleeding also initiates a pro-inflammatory effect on hypervascular and hypertrophied synovium [15]. The intra-articular processes of joint degeneration, capsular stiffening, and chronic inflammatory processes described in osteoarthritis [16,17] are likely very similar for PWH though yet to be fully investigated. Those PWH living with co-existing haemophilic arthropathy struggle to differentiate between pain associated with arthritic changes and acute onset joint pain which may or may not be associated with a bleeding episode [18]. This lack of specificity in perceived causal pain mechanisms is further complicated by wider medico-social, psychological, and cultural influences on the individual pain experience that accompany a rare inherited disorder such as haemophilia.

Bleeding and its management has been traditionally viewed within a model of biomedical intervention. Whilst this is appropriate within an acute bleed treatment episode or when engaging PWH in prophylaxis regimes to prevent bleeding, it struggles as a model of care provision to recognise the many competing experiences and influences which impact how an individual lives with their haemophilia. Pain associated with haemophilic arthropathy interferes with daily activity, mobility, work, and employment prospects [19,20]. PWH who have co-existing acute and chronic pain report a significant negative impact to their quality of life [21], which is made worse with multiple affected joints [22]. Recent UK NICE guidelines for chronic pain management encourage a more person centred approach [23] but there are limited accounts of the subjective exploration and attempts to understand the pain experiences of PWH. Knowledge and understanding of the life experiences of PWH in relation to haemophilia in their family, experiences of treatment and their own cultural and medico-social experiences may help enhance and inform approaches to pain management in the clinical context.

This study forms part of a larger project aiming to develop and test the feasibility of a rehabilitation based intervention as a component of a pain management approach for PWH. The development process sought to understand and explore the lived experience of pain in people with severe haemophilia, understand their beliefs around current pain management approaches and explore their views of exercise as a potential component of management, so as to inform and theoretically underpin a study protocol for a feasibility study. The current study aims to explore the life experiences of pain in people with severe haemophilia and understand how such experiences may influence their views and beliefs of pain now in adulthood.

## Methodology

### Study design

As the main aim of the study was to better understand the phenomena of pain experience in PWH, a qualitative approach using focus groups and semi-structured individual interviews was used.

With the focus of this study being the individual’s subjective reality of their life experiences and beliefs of haemophilia and pain, it was underpinned within a relativist ontology, situated in an overlapping phenomenological and interpretivist epistemology. Within a constructionist paradigm, meaning from the social and cultural world is constructed and a better understanding of what it is like to live with haemophilia and chronic pain is gained.

### Research team and reflexivity

The first author is a male clinical academic physiotherapist, 21 years qualified and with 15 years of experience of working in haemophilia, managing acute musculoskeletal bleed-related pain and observing the unique difficulties in managing chronic pain in this population. Beliefs about this study and approach were informed by the iterative development of the physiotherapy service over recent years in partnership with those PWH registered at the centre. Rigour and reflexivity throughout the study were maintained with regular research team meetings as well as a second author being present at the focus groups, and analysis and review of field notes and reflection. Analysis of data was discussed with the research team (one female nurse researcher, one female haematologist, two male academics, and one male person with haemophilia) and themes were discussed, modified reviewed and agreed.

### Recruitment

Participants with were purposively selected from southeast and northwest England in order to achieve a variety of views and experiences. Inclusion criteria were those with a diagnosis of severe haemophilia A or B, who self-identified as having persistent pain associated with their haemophilia, i.e., the presence of haemophilic arthropathy diagnosed from clinical assessment, aged 18 or over and with an absence of any other condition that would be responsible for the presence of persisting musculoskeletal pain. Those who could not provide consent, those with mild or moderate haemophilia or had pain not associated with haemophilic arthropathy were excluded.

The study was promoted on social media platforms as well as using posters designed in conjunction with the UK Haemophilia Society (patient organisation). Those interested were encouraged to contact the lead author by phone or email to initiate further discussions to clarify any queries as well as check inclusion criteria.

### Setting/location of groups

Two focus groups were held, in northwest and southeast England. Due to Covid-19 restrictions, telephone interviews were arranged in advance at the most convenient time for each individual participant.

### Methods

The focus groups and interviews took place over a nine month period between June 2019 and March 2020. Written informed consent was taken on arrival on the day of the focus groups and by email on the day of the telephone interviews. The study was approved by the St. Georges University of London Research Ethics Committee (reference no. 2018.0309). The study was not registered.

The meeting opened with an agreement of the attendees to the ground rules of confidentiality and respect for others voice and opinion within the discussions. A topic guide was developed in advance for the focus group with input from the research team which also includes a person with haemophilia, and was used to inform all the research questions developed for the larger overall study (see Supplemental information). Questions were based on the overall aims and objectives of the study. Questions were open ended to encourage and enable free conversation and an opportunity to raise issues and topics of concern to those present. The topic guide for the interviews was based on the same guide as the focus groups, with small adjustments being made to question style in acknowledgment of the 1:1 approach. Probing questions and prompts were also used in the groups and interviews to gain deeper understanding of experiences and views being discussed. The approach was flexible enough to enable and engage with topics and discussions that were introduced by participants as relevant to them.

Each focus group had two moderators from the research team present. The lead author led discussions in the group whilst the second moderator provided support in observation of participants, made field notes, and helped draw in quieter participants to the discussions as necessary.

Focus groups and interviews were digitally recorded and transcribed verbatim.

### Analysis

The analytic interest was focussed on the participants’ personal experiences of haemophilia and pain and so an approach using reflexive thematic analysis (TA) was deemed appropriate. Broadly, TA seeks to identify and analyse patterns in data and is a shared approach across other analysis approaches such as interpretative phenomenological analysis and grounded theory. Reflexive TA acknowledges the importance of the researcher subjectively as an analytic resource as well as a resource for knowledge production [24]. This interpretivist approach views the researcher as never being truly separate from their own values and beliefs [25] and so places my ontological view as that of recognising multiple realities (relativist) within a subjective epistemology.

The six phase approach to reflexive TA was used here, and is viewed as a recursive, iterative process rather than a truly linear one. The phases are described as (1) familiarisation with data; (2) generating initial codes; (3) initial theme generation; (4) reviewing and developing themes; (5) refining, defining, and naming themes and (6) writing up [24,26]. Transcripts were initially read alongside the recording of the interview, first to check for accuracy of transcription and then again as a way to begin immersion in the data. The lead author led the analytic process of coding and theme development. Coding within reflexive TA is not a process for finding pre-conceptualised themes, but instead is fluid process integral to theme development [27].

The concept of data saturation was not used here as an end point to coding and theme development. Instead, and in keeping with reflexive TA approach, we did not identify codes/themes *a priori* to data analysis [28]. The inductive approach used here represents situated and contextual engagement and interpretation of data by the lead author rather than using consensus between coders (i.e., codebook approach).

Initial interpretations of codes, broad theme development and thoughts about the data’s story were discussed with the other moderator who attended the group. As theme development became more solid, the wider research team discussed the findings leading to further refinement and analysis with codes being merged, removed, expanded and renamed as the data were further interrogated.

NVivo 12 Pro^®^ was used was used to manage the dataset (transcripts and field notes).

### Findings

A total of 14 people with haemophilia took part in this study. Sixteen people expressed an interest in taking part in the focus groups, but five were unable to attend on the specified date of the meeting. 11 PwH attended two focus groups (median age 52, range 28–73) and three were interviewed over the telephone (median age 28, range 23–30). Approximately, half of the participants were known to the lead author, as they were registered at the centre where he worked. Rather than bias, this familiarity was viewed as being beneficial to the process. Participants had a trusting relationship with the lead author, appreciating the need to be open and honest in the group/interviews and the importance of being able to share and discuss their experiences. The presence of a second moderator in the focus groups also ensured that reflexivity was strengthened in the analysis following. There were approximately 10 h of recorded interviews transcribed. Table 1 presents the demographic information of all those who participated. Pseudonyms are included for use in the narrative that follows. Two themes were conceptualised from the data: (1) haemophilia and pain – an evolving life biography and (2) “*My normal isn’t normal*” – identify and self-agency.

**Table 1.** Participant demographics (pseudonyms) – people with haemophilia.

	Pseudonym	Age	Diagnosis	UK/non-UK born	Employment	Prophylaxis
1	Tony	55	SHA	Non	Y	Yes
2	Adam	28	SHA	UK	N	Yes
3	John	42	SHA	UK	Y	Yes
4	Jack	57	SHA	UK	N	Yes
5	Greg	39	SHB	UK	Y	Yes
6	Will	52	SHA	Non	N	Yes
7	Ivan	73	SHB	UK	Retired	Yes
8	Alex	58	SHA	UK	Retired	Yes
9	Owen	52	SHA	Non	Y	Yes
10	Andy	40	SHA	UK	Retired	Yes
11	Hugh	65	SHA inhibitor	Non	Y	Yes
12	Sean	23	SHA	Non	Student	Yes
13	Leon	28	SHB inhib	UK	Y	Yes
14	Nick	30	SHA	UK	Y	Yes

SHA: severe hemophilia A; SHB: severe haemophilia B; inhibitor: (presence of antibodies that prevent factor replacement treatment from working effectively).

#### Theme 1: haemophilia and pain – an evolving life biography

Here the medical, historical, and social evolution of life with haemophilia is explored, particularly relating to living with a rare disorder and the pain associated with it.

##### The historical narrative of haemophilia

For older men with haemophilia, early life experience with medical care of haemophilia is primarily reflective on a lack of any medical treatment and limited awareness of haemophilia. Significant periods of hospitalised immobilisation was normal, and invariably ended up provoking further bleeding. In early years a lack of treatment and expertise from both parents and the medical profession meant that being cared for was viewed as “*damage limitation*” *(Hugh, 65)*. For almost all older participants, “excruciating” pain from bleeding was a repeated and common experience of growing up with haemophilia:

I remember one incident where I was on admission and I had multiple anal inserts, and unfortunately once you get one in they can’t give you another for a couple more hours. At the end of the day, they had to resort to morphine shots to calm me down, and even that couldn’t keep me calm for, like, 15 to 20 minutes before the pain starts to shoot through the roof again. (Andy, 40)

Localised non-specialist medical care and potentially dangerous or ineffective interventions for pain relief were the norm until development of, and access to effective medical treatment revolutionised acute bleed management.

This must have been in the early ‘60s, I think – they gave me one of the first experimental doses of the factor IX concentrate, and that worked. And I thought, “WOW!” … Because that fairly quickly stopped the bleed and the pain went down. (Ivan, 73)

As care advanced from hospital based treatment to clotting factor concentrates administered independently at home, people with haemophilia began to see positive enhancements to their life and activity. The relationship between the symptom of pain and the possibility of that pain being a bleed is complex and has evolved over time and with age, experience of treatment improvements and treatment availability. However, the advent of effective prophylaxis and diminishing episodes of spontaneous bleeds is challenging the internal reasoning and decision making processes that uses pain as a marker for bleeding-

I suppose you don’t really know the difference, because you would always associate a joint bleed or joint pain with a bleed. (Adam, 28)

##### Haemophilia, pain, and the family

The diagnosis of haemophilia within a family was for some parents a catastrophic disruption to life. A child with haemophilia meant that they were required to blindly navigate imperfect medical care as well as trying to provide the best parental care and protection they could, at a time when knowledge and medical provision were scarce:

… diagnosed at six weeks old – I think, from memory – it came as a complete shock to my parents. They’d never heard of haemophilia or how to cope with it, or how to manage me. I was their son. I was in incredible pain a lot of the time. (Ivan, 73)

Living with haemophilia and the bleeding episodes was disruptive to family life, familial roles, and relationships. The child with haemophilia brought with it responsibilities and need for management that was more than what would be seen normally within most families at the time. Anxiety of having to disclose a bleed to a parent for fear of an angry response, resulted in many hours of no treatment and increasing pain:

And my dad was like really strict, so what I’d do is, if I hurt myself, I’d try and treat myself, like get… like try and… “Oh God, I’ve got to tell him, I’ve got to tell him.” And then the ambulance would come, like half eleven at night, and I’d make it worse, and I’d have to be in hospital. (Jack, 57)

Pain associated with haemophilia was an ever present and normalised feature within the family unit whilst growing up. How participants experienced, reported, and behaved with pain has evolved into their adult lives, continuing to be a feature in their own families now. Some feel that their pain is a personal experience not to be shared or discussed with their partners or family, whilst others describe teaching vigilance of their pain to their children:

If it’s a bad day, I will try and keep my son aware to some degree, so that he can understand if I’m snappy it’s not because of something he’s done. It’s difficult because he’s only eight… he knows his dad has issues in certain areas. (Will 52)

##### Fear, consequence, and adaptation

The worry pain may be a possible sign of bleeding permeates all cognitive processes, behaviours, and activity choices in the lives of men with severe haemophilia –*‘The last thing you want is a bleed – that’s the thing to avoid.’ (Tony, 55)* Whilst pain is unpleasant, it is an experience that is accepted as almost always being present and is to a degree, accommodated and moderated. Bleeding, although intermittent, is unpredictable and has greater consequences on immediate and future physical ability and social interactions.

I’m just sort of thinking, at the back of my mind I’m hearing pain is like half an inch away from… not a bleed, but disaster. You know, because you might not just have a bleed – like a bad knee, a bad arm or something. I’ve been hospitalised with a psoas bleed and I was lucky to stand up straight after that. (Hugh, 65)

The fear of bleeding has lessened with being on prophylaxis. Fear and anxiety as a response is still manifest, but more in relation to the consequences of their lifetime of haemophilia on their physical, social, and psychological being. Worry about bleeding merges with the constant stress of monitoring the state of their joint on a daily basis:

…with the joint pain for me: there’s a degree of unpredictability. Some days you can just push through and yes, you may be sore the next morning, but you will be okay. And other days, you’re going to be in a lot of pain that night. (Will, 52)

Haemophilia and its physical side effects is viewed as something that prevents an acceptable, predictable continuity of life. The need for a structural cause to attribute to pain is coherent with a biomedical model of health and well-being that has been a feature of the medical care approach and life with haemophilia. The presence of pain independent of bleeding or injury is a difficult concept for many to contend with, presenting a challenge in what to do, embedding doubt and the possibility of negative consequence and inhibiting physical activity even more:

You’re constantly in a protective mode, aren’t you, really. (Tony, 55)

#### Theme 2: “My normal isn’t normal” – identity and self-agency

Here, we present how the sense of physical self, both that observed by others and the internalised perception of bodily and social identity because of haemophilia has, and continues to be, influenced by internal and external factors.

##### Physicality and ableism

The bodily and perceptual changes acquired because of haemophilia start in early age. The internal sense of physical self exists alongside ongoing salience for bleeding, alongside comparison of themselves against unaffected peers. Reflection and reminiscence of being younger and what was “*their best years*” *(Andy, 40)* is common, often related to feelings of having been more active and having less pain, but viewed now as a loss because such enjoyment with activity is fleeting and unlikely to be achieved with their current physical state. Whilst the loss is mourned it is also described in terms of inevitability in having to make decisions to stop enjoyable physical activities as *“it was more important to be fit and able to go to college the next day, or to go to my workplace, or got to my Saturday job” (John, 42)* than risk bleeding and more pain.

Men with haemophilia appear to want to be able to do more despite their physical limitations. They want support to do so, acknowledging the difficulties are physical, practical, and psychological because getting older with affected joints is hard and brings with it other issues of physicality and *“there’s no point in living longer if you’re in a mess” (Hugh, 65).*

Even with support, issues with body image and environments that do not accommodate disability impair physical activity and so trying to be physically active becomes more difficult:

You see, I enjoyed swimming, but then I had trouble getting out of the pool. And then there’s the embarrassment factor… trying to get out of the pool. So the arm is bent, I can’t push myself up, and then trying to get out up the steps, I’d hold the steps but I’d have to make sure I’ve got my feet planted just right so I can pull up on that. (John, 42)

##### Difference and sameness

Perceptions of identity in PWH are complex, existing in the social setting as well as implicit in their search for comparators to them and normalcy in day to day life. Exposure to such views happens early on at school, with their differences and social capital being negatively influenced by others in positions of authority-

I remember, the first day of secondary school, I was brought up in front of assembly, in front of 300 kids, and pointed at, and they said, “Don’t touch this guy.” The first day of my first year at secondary school, they said, “Can Jack please come to the front, please.” The first day at this new school. “This boy, he’s delicate and he bleeds.” (Jack, 57)

Whilst some avoided social contact that carried any risk of bleeding or injury, others were removed from school entirely to be home schooled, further highlighting their difference. There is a complex relationship with past experiences and how it influences social identity with haemophilia, to the point where it has negatively influenced behaviours that could have been of benefit:

I live in a really big student town. I joined [the gym] in July or August and all the students were away and it was marvellous. It was great… having that kind of window of opportunity to go and explore and begin to feel comfortable and use the machines and just having a play around, particularly having never felt like I belonged in those kinds of spaces, was really useful. (Nick, 30)

As well as social identity perceptions there appears to exist a multilayered view of identity specific to haemophilia itself. Acceptance of the condition and its effects by both HCP’s and broader society is important, as being a person with haemophilia in itself is not how these men want to be defined, although there is acceptance that as a group “*the legacy is we have been damaged*” (Hugh, 65).

Upward comparison to those perceived as normal appears to help strengthen their own perception of self with haemophilia, particularly in regards to pain its intensity and their ability to cope with it, and the view that PWH have “*experienced real pain*” *(Owen, 52)*.

There is a downward comparison made also, in that those without haemophilia who have poor surgical recovery, for example, just did not work hard enough. This appears to relate to a perception that PWH have developed a better fight to work harder to recover, because they have had to do it so many times. Pain is as much part of the identity of a PWH as having haemophilia. Pain is normal and life experiences embed the acceptance of pain within their view of themselves.

Whilst examples of upward and downward comparison appeared to be used to strengthen self-perceptions, comparing self with other PWH raised more uncertainty and questions about their own views and behaviours. There is judgement and disbelief about those with haemophilia who can participate in elite sporting activity – *“We’ve got an extremist in our membership – Alex Dowsett” (Owen, 52)*. In a modern era of better haemophilia treatments, there appears to be a constant challenge to their view of themselves leading to questions about if they should and are able to do more. Individual fears about pain and possible negative effects are confronted and challenged by seeing and hearing about others with haemophilia having some positive outcomes with activities such as exercise, further challenging perceptions of identity.

## Discussion

The aim of this study was to investigate and explore the life experiences of pain in PWH and to understand how such experiences may influence their beliefs and sensations of pain now in adulthood. The account presented here highlights that pain for PWH is a lifelong, continually evolving experience that has been deeply influenced by social, cultural, and medical experiences within that lifetime. It is this novel qualitative exploration and explication of the multifactorial influences on pain in PWH that provides findings of potential clinical relevance.

To contextualise an understanding of the pain experience in PWH, it is important to understand the historicity of such experiences within a timeline of medical treatment. Up to the 1950s/60’s management of acute musculoskeletal bleeding was limited to bed rest and access to transfusions of whole blood or fresh frozen plasma [29]. This treatment was scarce, came with a substantial treatment burden and discomfort, and was only prescribed if the doctor deemed the bleed severe enough, which meant many PWH avoided it by staying at home and tolerating the pain of bleeding. Pain associated with acute bleeding was seen as something to be managed in co-ordination with bleed management and resolution, particularly with the development of effective treatment in the 1970s, whilst chronic pain presented “considerable therapeutic difficulty” [30] with advanced haemophilic arthropathy management viewed as a palliative endeavour [31].

The consequences of childhood pain on parental emotions are also challenging, and those in this study were all very aware from a young age how their condition affected their family. Managing unpredictable challenges such as bleeding and pain for mothers of boys with haemophilia was beset with distress [32]. Similarly, parents of children with Juvenile Idiopathic Arthritis report desperation in trying to manage painful episodes associated with the illness and the physical and emotionally draining effect on the entire family and family life being affected [33]. The fear of a child being in pain and the ever present feeling of potential danger traps parents in a “cage of fear”, resulting in an ingrained behaviour of always thinking about possible consequences [34]. The constant surveillance for pain and danger is not only confined to parents. Young people with sickle cell disease report that they are always monitoring for signs of sickle crisis and pain so as to be ready to take action with it, but are mindful as to when and who to disclose it to so as not to be marked out as different or disrupt their life [35]. Such a view meant that non-critical sickle pain was often managed at home outside of any clinical context [36]. Palermo and Chambers [37] proposed an integrated model of factors relating to a child’s pain within the family. It highlights the reciprocal influence of pain itself on relationships, parenting styles/behaviours and family functioning and that such factors are not fixed as they depend on the age developmental stage of the child. Such a model helps contextualise the almost constant requirement of parents of children with haemophilia in trying to manage recurrent painful bleeds in an era of limited medical treatment. It is notable that there are similarities in this parental behaviour seen now in adults with haemophilia. Whilst they contemplate their pain and the ever present fear of bleeding, how they choose (or not) to communicate about their pain also impacts on their own family as they try and manage their perceived burden of themselves on others.

Physical activity, due to its associated risks with bleeding, was curtailed in the formative years of many PWH. Whilst this imposed difference was unwelcome and stigmatising, many PWH still feel guilt that their own actions at the time have added to their current experience of pain and functional difficulties [38]. Rolstad [39] found in their qualitative study that older men with haemophilia carry a psychological burden that is influenced by the degree of social stigma and ignorance they encountered in their formative years. Whilst the cohort in this study are able to recount negative experiences of their life and pain associated with their haemophilia, it appears to be situated within a coherent, reasoned and somewhat positive account of that life – and one which makes sense to them and others with shared experiences. This is perhaps an extension of the coping strategies developed in childhood but it may reflect acceptance of what is felt to be currently realistic. A large ethnographic study of PWH in five countries, highlighted that although there is a trade off with pain and the need to stay active, there is a view for many that things used to be worse and that this perhaps prevents many from being able to live their fullest life possible. This lived experience of what was, alongside their individually appraised experiences of everyday life continue to influence how pain, function and activity exist within a desire to avoid bleed provocation at all costs [40].

Whilst living with a rare congenital condition can bring with it a burden of disease management, for many people they do not wish to be defined by that condition. Similar to findings by described by Kalmar et al. [41], participants in this study spoke with clarity on how their haemophilia does not define them and that living with haemophilia is their “normal” – for it is all they have ever known. The need for an illness or condition to not dominate life is highlighted in other conditions such as sickle cell disease, where individually perceived normalcy is constructed apart from the disease itself [42]. Likewise, the ability to accept and live with pain as they do was associated with the belief that PWH live with an enhanced pain tolerance due to their many previous experiences of pain, a finding also reported previously by others [43,44]. It is the association and identity of having pain alongside their haemophilia that is a notable exception in this study. Our data suggest that for many PWH their identity as a person with chronic pain is as much a part of their identity as haemophilia is. Whilst some broad similarities were found in our data, many of the men interviewed here appeared to be accepting of what they were able to do with their pain being present and they had altered their lifestyle accordingly. It is unclear if this is particular to this group of men included in this study, or perhaps a wider indication of access to both haemophilia treatment and experienced healthcare teams.

Understanding the individual, lived perspective of a PWH experiencing multiple painful events provides a better way of understanding their acute and chronic pain. It is the embodied relation between the person and their environment that helps shape the many different ways pain can be experienced [45], and therefore requires more than a unimodal biomedical approach. Acute pain (such as that in an acute bleed) captures attention by interrupting activity, demanding a response so as to protect the body part (as necessary), as well as creating a motivational context in which to do something (such as rest or take treatment in the case of bleed). The experience of chronic pain (as with joint arthropathy) continues to interfere with activities and the sense of self, and requires accommodation to the ongoing pain if a person is to define who they want to become with that pain [46]. The companion study to the one described here identified the limitations with current pain management approaches that PWH have experienced and highlights the uncertainties with unimodal pain management approaches such as exercise [47]. Whilst acknowledging local anatomical and higher cortical physiological responses to acute and chronic pain, we believe this current study provides much needed contextual insight in respect of possible contributing factors to the pain experience for people with severe haemophilia.

Despite the strengths of this study, there are acknowledged limitations. All of the participants included in this study were resident in the UK and therefore receiving what would be considered world leading haemophilia care. We acknowledge that those PWH in low resourced countries and in healthcare systems with limited or no access to effective haemophilia treatment may not have the same experiences or beliefs expressed by those included here. A strength, however, is that some of our participants did not grow up in the UK and so were able to express and discuss their experience of pain from that perspective. Further research should be mindful of the socio-economic and cultural influences of healthcare on the individual experience of pain in PWH.

This study only included people with severe haemophilia. This is in no way to diminish the experiences of those with moderate or mild haemophilia, but it remains that those with severe haemophilia remain most likely to experience more episodes of bleeding and painful joint damage from a young age. It is imperative therefore to understand these experiences. Further research should seek to explore if people with moderate and mild haemophilia have similar or differing life experiences of pain as well as thoughts and beliefs relating to pain.

A researcher led, reflexive TA approach rather than that of codebook approach to coding was employed here. This may mean other researchers who would come to analyse this dataset with a different philosophical position or clinical experience may come to different conclusions. This in itself is not a limitation but we present the positionality of the authors in the study and acknowledge that it is the unique clinical and academic experience of the lead author that enables the depth of analysis presented here.

## Conclusions

Pain is a well-established feature of acute bleeding in haemophilia. This study highlights that early experiences of bleeding and persistent pain associated with joint arthropathy may play a role in how pain becomes embedded in the life experience of living with haemophilia. Continuing to view pain as a wholly biomedical construct fails to appreciate and understand the effect of multiple unique pain experiences that PWH live with. Healthcare professionals in haemophilia should be mindful of the individual’s lifetime experience of pain in clinical encounters and seek to understand its relevance to practice and any interventions that may be required.

## Supplementary Material

Supplemental information
